# Lipid-Encapsulated Engineered Bacterial Living Materials Inhibit Cyclooxygenase II to Enhance Doxorubicin Toxicity

**DOI:** 10.34133/bdr.0038

**Published:** 2024-06-25

**Authors:** Ning Jiang, Wanqing Ding, Xiaojuan Zhu, Jianshu Chen, Lin Yang, Xiaoping Yi, Yingping Zhuang, Jiangchao Qian, Jiaofang Huang

**Affiliations:** ^1^ State Key Laboratory of Bioreactor Engineering, East China University of Science and Technology (ECUST), Shanghai 200237, China.; ^2^ Shanghai Collaborative Innovation Center for Biomanufacturing (SCICB), East China University of Science and Technology, Shanghai 200237, China.; ^3^ College of Life Science, Jiangxi Normal University (JXNU), Nanchang 330022, China.

## Abstract

Recently, there has been increasing interest in the use of bacteria for cancer therapy due to their ability to selectively target tumor sites and inhibit tumor growth. However, the complexity of the interaction between bacteria and tumor cells evokes unpredictable therapeutic risk, which induces inflammation, stimulates the up-regulation of cyclooxygenase II (COX-2) protein, and stimulates downstream antiapoptotic gene expression in the tumor microenvironment to reduce the antitumor efficacy of chemotherapy and immunotherapy. In this study, we encapsulated celecoxib (CXB), a specific COX-2 inhibitor, in liposomes anchored to the surface of *Escherichia coli* Nissle 1917 (ECN) through electrostatic absorption (C@ECN) to suppress ECN-induced COX-2 up-regulation and enhance the synergistic antitumor effect of doxorubicin (DOX). C@ECN improved the antitumor effect of DOX by restraining COX-2 expression. In addition, local T lymphocyte infiltration was induced by the ECN to enhance immunotherapy efficacy in the tumor microenvironment. Considering the biosafety of C@ECN, a hypoxia-induced lysis circuit, pGEX-Pvhb-Lysis, was introduced into the ECN to limit the number of ECNs in vivo. Our results indicate that this system has the potential to enhance the synergistic effect of ECN with chemical drugs to inhibit tumor progression in medical oncology.

## Introduction

In recent years, live bacteria have received increasing attention in the field of tumor treatment due to their unique ability to colonize tumors [1]. Live bacteria can consume nutrients within the tumor microenvironment, resulting in nutritional deficiency in tumor cells [2]. In addition, they can produce toxic factors, including bacterial toxins, which induce tumor cell apoptosis and inhibit tumor growth. However, the efficacy of bacteria-based tumor treatment strategies in clinical trials has been suboptimal. For example, *Salmonella* has shown strong antitumor effects in vitro, but experiments have demonstrated strong toxic side effects and weak therapeutic effects in vivo, failing phase I clinical trials [3]. Nevertheless, live bacterial materials continue to be a focal point in tumor treatment research due to their advantages and features such as active targeting [1,4], tumor colonization [5,6], and potential as genetic engineering chassis [7–9]. To further enhance the application of bacteria in tumor treatment, various live bacterial materials have been developed, such as surface modification [10–12], chemical encapsulation [13,14], and molecular modification to express functional proteins [15,16]. These advancements have yielded significant improvements in antitumor outcomes. However, the *Escherichia coli* Nissle 1917 (ECN)-induced inflammatory response consistently triggers the up-regulation of cyclooxygenase-II (COX-2) in tumor cells through interferon, interleukin-12, and other cytokines [1,17–19]. COX-2 also promotes the expression of the downstream prostaglandin E2 (PGE2)/Bcl-2 axis [20]; enhances the antiapoptotic ability of tumor cells; and reduces the therapeutic effects of chemotherapy drugs, photodynamic therapy, and radiotherapy [7].

COX-2 is a rate-limiting enzyme that catalyzes the production of PGE2 from arachidonic acid and is expressed at the tumor site [21]. Multiple studies have confirmed that the tumor inflammatory tumor microenvironment can activate the axis of COX-2/PGE-2/Bcl-2 pathway, thereby activating the expression of the antiapoptotic gene Bcl-2 in downstream tumor cells and enhancing the antiapoptotic ability of tumor cells [22]. Therefore, COX-2 inhibition-based antitumor treatment strategies have been widely developed in recent years [23]. Celecoxib (CXB), a nonsteroidal anti-inflammatory drug, specifically inhibs of COX-2 expression [24,25]. However, prolonged usage gives rise to adverse effects such as constipation and vomiting, limiting its application in tumor treatment. Although nanodrug delivery systems (liposomes [26], albumin [27,28], etc.) can overcome some of these obstacles to a certain extent, clearance by the reticuloendothelial system in the liver and the high-density tumor extracellular matrix still results in an overall utilization rate of less than 0.7% [29]. To overcome this predicament, various targeted modification drug delivery systems, such as AS1411-modified liposomes and ADC drugs, have been developed [30,31]. However, complex tumor heterogeneity and surface antigen loss ause these delivery strategies to lack broad spectrum and potential off-target effects. As one of the characteristics of solid tumors, the hypoxic tumor microenvironment can selectively recruit anaerobic microorganisms for colonization [32,33]. Therefore, the development of tumor treatment strategies based on anaerobic microorganisms for drug delivery holds great promise in the field of oncology.

To overcome these issues, we introduced the hypoxia lysis circuit, pGEX-Pvhb-Lysis, into ECN to establish the safe drug delivery platform, C@ECN-PL, for the treatment of tumors [34–36]. First, we modified the surface zeta potential of ECNs using glycol chitosan (GC). C@ECN-PLs were prepared by encapsulating CXB in liposomes through a thin-film hydration method and then by adsorbing it onto the surface of the ECN. C@ECN-PLs enhance drug utilization to reduce the toxicity of CXB and inhibit tumor resistance caused by high expression of COX-2 in tumor cells. In vivo experiments have shown that C@ECN-PLs can specifically colonize at the tumor site, increasing the accumulation of CXB in the tumor site by approximately 5-fold. Moreover, C@ECN-PLs effectively inhibited the expression of COX-2 at the tumor site and enhanced the sensitivity of tumor cells to chemotherapy drugs. Subsequently, we combined doxorubicin (DOX) with C@ECN-PL, and the experimental results showed that C@ECN-PL significantly enhanced the antitumor effect of DOX, inhibited tumor cell growth, and prolonged the survival period of mice in vivo. In summary, the C@ECN-PL system developed in this work not only has good biocompatibility and specifically enhances the accumulation of drugs at the tumor site to improves drug utilization, providing a new strategy for the further application of ECNs in clinical settings.

## Materials and Methods

### Preparation and characterization of CXB@Lipo and C@ECN

CXB@Lipo (C@L) was obtained by the thin-film hydration method. Briefly, CXB (1 mg), cholesterol (38 mg), and HSPC (200 mg) were dissolved in chloroform. Then, chloroform was removed from this hybrid solution by a rotary evaporator from the film on the round-bottom flask. Next, the film was retuned by adding 2 ml of H_2_O and sonicating for 10 min. Following this, the pre-C@L was further extruded with a liposome extrude (LiposoFast, Avestin, Canada) through 200-nm polycarbonate membrane filters (Merck Millipore Ltd., USA). Finally, the prepared C@L was concentrated in an ultrafiltration centrifuge tube (30 kDa at 2,000 rpm for 30 min).

C@L was riveted on the surface of the ECN, ECN (1 × 10^7^) was resuspended in PBS buffer, and 1 mg of glycol chitosan was added and stirred for 30 min. Then, 1 mL of C@L was added to this buffer (ECN-GC) and stirred for another 30 min. Finally, C@L was removed by washing the complex 3 times with H_2_O and the mixture was stored at 4 °C for 1 week.

The morphology of the ECN or C@ECN was determined via transmission electron microscopy (TEM; JEM-1400, Japan). The average size and zeta potential of the various groups were measured by a Malvern Nano ZS ZEN3600 (Malvern, UK). The characteristic absorption of CXB, liposome, and C@ECN-PL was also determined by UV-VIS (Thermo Fisher Scientific, USA). ICG was used to replace CXB using the above method to obtain ICG@ECN (I@ECN). Then, the effect of mixed liposomes loaded with I@ECN was tested by flow cytometry (CytoFLEX, Beckman, USA).

The viability of C@ECN was determined in LB media supplemented with UV‒VIS. Then, 10 μL of ECN, ECN-GC, or C@ECN was added to 5 mL of LB media and incubated at 37 ℃ and 200 rpm. The number of ECNs was determined by the absorption at OD_600_ nm using UV‒VIS.

### Preparation of C@ECN-PLs

pGEX-Pvhb-Lysis was constructed through the Gibson self-assembly method, involving cleavage of the pGEX plasmid by Bam HI and Eco RI endonucleases, seamless ligation of the Pvhb-Lysis gene fragment into linearized pGEX using a cloning kit (Beyotime Biotechnology Co. Ltd., Shanghai, China), and subsequent introduction of the ligated plasmid into ECN competent cells.

### Cell uptake of C@ECN

To determine the uptake effect of C@ECN or C@L, ICG was used to replace CXB with ICG@Liposome (I@L) or I@ECN [ICG concentration of 0.1 mg/ml and 10^8^ cfu of ECN], respectively. CT26 tumor cells (1 × 10^6^) were seeded into 30-mm glass plates. After incubating for 12 h, fresh DMEM containing I@ECN or I@L was added to the CT26 tumor cells and incubated for another 4 h. Then, the DMEM was removed, and the cells were washed with PBS 3 times. The sections were incubated with DAPI for another 10 min at room temperature, washed with PBS 3 times and stored at 4 °C. Finally, the cells were imaged by confocal laser scanning microscopy (CLSM) (Nikon, Japan).

### Cell cytotoxicity assay

CT26 and MCF-7 tumor cells were seeded into 96-well plate at a starting concentration of 10^4^ cells per well. After incubation for 12 h, free DMEM containing various concentrations of C@L, ECN, C@ECN-PL, C@L + DOX, ECN + DOX, or C@ECN-PL + DOX was added to the medium (DOX concentration of 1.5 μM) and the cells were incubated for another 24 h. Finally, cell viability was determined with a CCK-8 assay kit (Beyotime Biotechnology Co. Ltd., Shanghai, China).

### COX-2 expression analysis

To determine the expression level of COX-2 after various treatments, CT26 tumor cells (10^6^) were seeded into 6-well plates, respectively. After incubating for 12 h, the cell were incubated with fresh DMEM supplemented with 20 μM CXB for another 12 h. Then, RIPA lysis buffer was used to extract proteins from these cells. The expression levels of COX-2 and β-actin were determined by Western blot (WB) analysis. Additionally, all these images were further analyzed and quantified using ImageJ.

CLSM was also used to further test COX-2 expression. Briefly, CT26 or MCF-7 tumor cells (10^6^) were seeded into 3cm glass plates. After 12 h of incubation, 20 or 30 μM CXB was added to the plate, and the cells were cultured for another 6 h. Then, the DMEM was removed, and the cells were washed 3 times with PBS. The cells were stained with a FITC-conjugated anti-COX-2 antibody (Affinity, Jiangsu, China) for 2 h at 4 °C, followed by DAPI staining for another 10 min and 3 washes with PBS. Finally, CLSM was used to test the fluorescence signal. After CT26 cells were treated with the aforementioned method, they were collected in 1.5 mL EP tubes and subjected to flow cytometric analysis.

### Pharmacokinetics and biodistribution of C@ECN in vivo

The biodistribution of C@ECN was investigated using an in vivo images system (IVIS). In total, 10^7^ CT26 cells were subcutaneously injected into the left forelimb under the armpit of 6-week-old BALB/c mice. Once the tumor volume reached approximately 100 mm^3^, the mice were randomly divided into 2 groups and intravenously administered I@L or I@ECN (ECN, 10^6^). The biodistribution of I@L or I@ECN was observed through imaging at 0, 12, 24, 48, and 72 h after injection. After the 72-h treatment period, the mice were euthanized, and major organs (heart, liver, spleen, lung, kidney, and tumor) were collected for in vitro fluorescence signal testing.

Tumor tissues were harvested and subsequently embedded in optimal cutting temperature (OCT) compound. After treatment with I@L or I@ECN for 48 h, the specimens were sectioned into slices of 10 μm thick slices. After incubation with DAPI for a period of 10 min, the slices were imaged via CLSM to capture their fluorescence characteristics.

### Antitumor efficacy of C@ECN-PLs in CT26 tumors in vivo

CT26 tumor model was established by subcutaneous transplantation. First, CT26 tumor cells (10^8^) were injected into the left forelimb under the armpit of BALB/c mice to establish the main tumor model. When the tumor volume reached approximately 100 mm^3^, the mice were randomly divided into 4 groups: PBS, DOX, CXB + ECN-PL + DOX, and C@ECN-PL + DOX, respectively (DOX, 4 mg/kg; CXB, 5 mg/kg; ECN, 10^7^ CFU). Tumor volume and body weight were measured by a Vernier caliper and electronic scale every 2 d for 14 d. The tumor volume was calculated by the following formula: (major axis) × (minor axis^2^)/2. After 14 d, the mice were sacrificed, and tumors were collected for weighing.

### Evaluation of tumor apoptosis and proliferation in vivo

The CT26 tumor model was established by the above method. All the mice were sacrificed, and the tumor tissue was collected and stored in OCT gel and cut into 6-μm slices at −20 °C. First, this slices were blocked with PBS containing 5% FBS for 30 min. Then, FITC-conjugated anti-mouse CD3, FITC-conjugated anti-mouse CD4, and PE-conjugated anti-mouse CD8 were used to stain the slices for 4 h (dilution factor 1:500, Biolegend Inc., USA). After staining with DAPI for another 10 min and washing with PBS 3 times, the fluorescence signal was obtained by CLSM. In addition, TUNEL and H&E staining were used to analyze CT26 tumor apoptosis in vivo, and Ki67 was used to assess proliferation after various treatments.

### Biosafety evaluation of C@ECN-PL

The biosafety of C@ECN-PL was evaluated by intravenously injected into BALB/c mice. Briefly, healthy mice were intravenously injected with C@ECN-PL at a concentration of (5 mg/kg) CXB, and the number of ECNs was (10^7^ CFU). Seven days later, the mice were sacrificed and blood and major organs (heart, liver, spleen, lungs, and kidney) were collected. The serum levels of aspartate aminotransferase (AST), alanine aminotransferase (ALT), blood urea nitrogen (BUN), and creatinine (CRE) were evaluated by assay kits purchased from the Jiancheng Bioengineering Institute (Nanjing, China). The integrity of major organs was also evaluated by H&E staining analysis, and images were further quantitatively analyzed by ImageJ 6.0.

### Statistical analysis

Statistical analysis was performed via a one-way analysis of variance (ANOVA) test. Moreover, post hoc analysis was performed using the Wilcoxon rank sum test with a Bonferroni correction when needed. *P < 0.05 was considered to indicate statistical significance; **P < 0.01 and ***P < 0.001 were considered to indicate extreme significance; NS, no significant difference.

## Results and Discussion

### Preparation and characterization of C@ECN-PLs

**Fig. 1.**
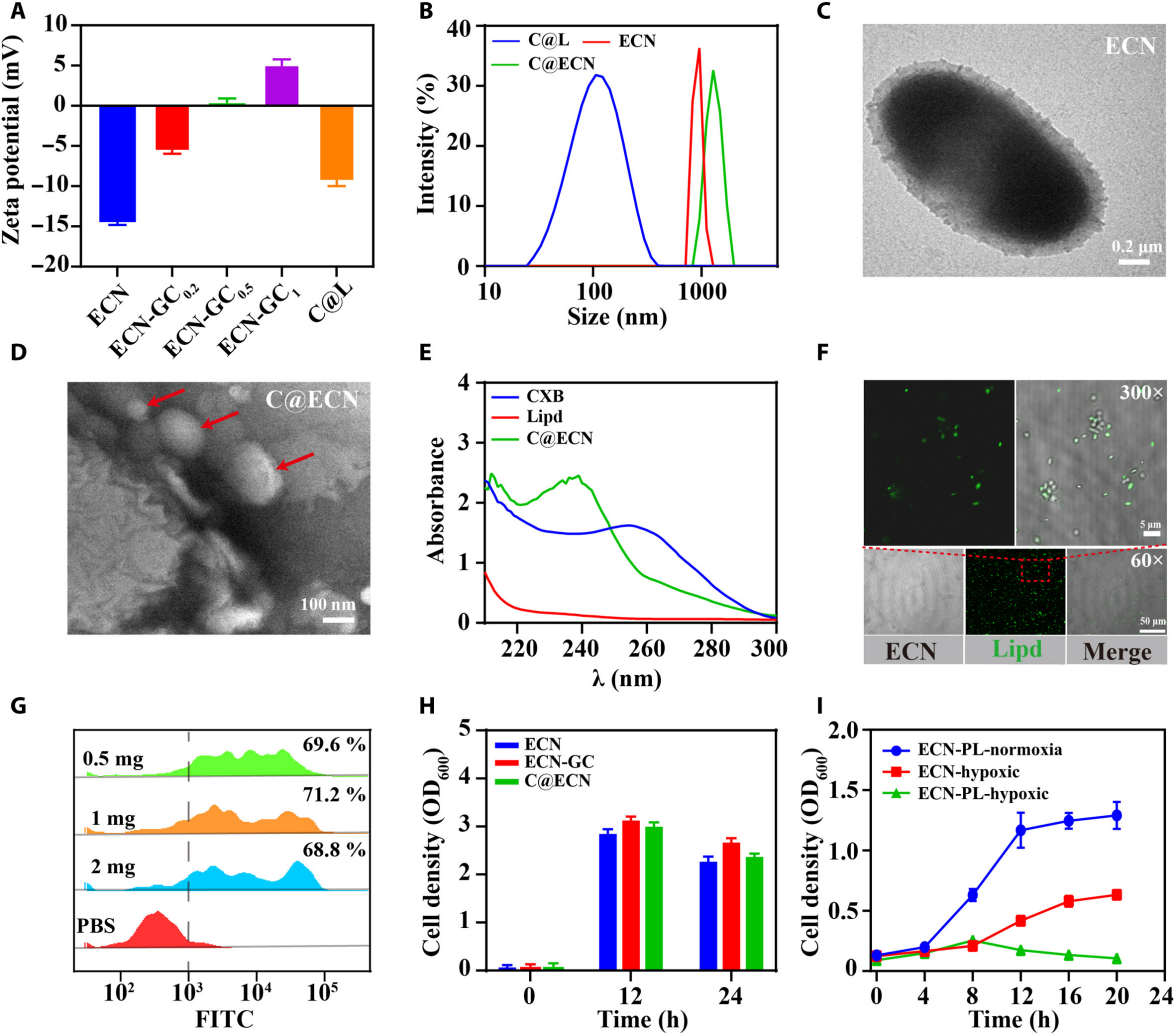
Characterization of C@ECN-PLs. (A) Changes in the zeta potential of ECN incubated with different concentrations of GC. (B) Changes in the size of ECNs attached to liposome. (C and D) Representative TEM images of the ECN strain and C@ECN; C@L is marked by a red arrows. (E) UV-VIS spectra of free CXB, liposomes, and C@ECN in a PBS buffer. (F) Representative fluorescence images of C@ECN. (G) The loading efficiency of liposomes onto the surface of ECNs. (H) Growth behavior of ECN, ECN-GC, and C@ECN in LB medium. (I) Growth of ECNs or C@ECN-PLs in different environments.

To successfully anchor negatively charged liposomes on the surface of the ECN, we first modified the surface charge of the ECN using various concentrations of GC. Because of the abundance of amino groups in chitosan, GC adsorption onto the ECN surface can alter its surface charge to a positive potential. As shown in Fig. 1A, the zeta potential of the ECN surface shifted from negative (−14.8 ± 0.6 mV) to positive as the concentration of incubated GC increased. When the GC concentration reached 1 mg/mL, the zeta potential of ECN shifted from −14.8 to +5 mV. Subsequently, liposomes were introduced to ECN-GC through electrostatic interactions, leading to the attachment of liposomes onto the ECN surface and resulting in a significant shift in the zeta potential of ECN from +5 to −8.9 mV. The change in the size of the ECN before and after liposome attachment was measured using a Malvern particle size analyzer (Figure 1B). The size of liposome@ECN (1.2 μm ± 0.2 μm, denoted as L@ECN) was larger than that of ECN (960 nm ± 20 nm), suggesting the potential adsorption of liposomes onto the surface of ECN, leading to an increase in the size of L@ECN. To further confirm the electrostatic adsorption of liposomes onto the ECN surface, the morphology of L@ECN was examined using TEM. Figure 1C-D clearly shows the presence of liposomes surrounding the ECN, indicating the successful loading of liposomes onto the ECN surface. CXB@Lipo@ECN was prepared following the above method (referred to as C@ECN), and UV‒VIS spectroscopy was employed to analyze the characteristic UV absorption of CXB. As shown in Figure 1E, free CXB exhibited a distinctive absorption peak at 256 nm, whereas C@ECN displayed characteristic absorption at 240 nm. This observed blueshift phenomenon could be attributed to the influence of liposome encapsulation on CXB. To ensure the formation of a stable structure in C@ECN, the colocalization of ECN and liposomes was determined using the lipophilic dye DIR as a substitute for CXB and encapsulated within the liposomes. Figure 1F shows the colocalization of green fluorescence with ECN, indicating that CXB can be encapsulated onto the ECN surface, resulting in the formation of stable C@ECN. To determine the maximum loading ratio of C@L on the ECN surface, different concentrations of DIR@Lipo were added to the ECN and subsequently analyzed using flow cytometry (Figure 1G). The results revealed that the maximum loading ratio was achieved when the liposome concentration reached 0.5 mg/mL. Even with the addition of 2 mg/mL liposomes, the surface coverage rate of the ECN remained at 68.8% ± 2%. Because the ECN has an active tumor-targeting capability, the viability of C@ECN was evaluated, as illustrated in Figure 1H, revealing no influence on the activity of the ECN following the attachment of C@L. This suggests that C@ECN exhibits a remarkable capacity for active targeting of tumor cells based on the ECN in vivo. Furthermore, to ensure the safety of ECN in vivo, an oxygen-induced controlled release circuit, pGEX-Pvhb-Lysis, which incorporates a promoter, Pvhb, capable of sensing the hypoxic tumor microenvironment and controlling downstream gene expression, was integrated into the pGEX plasmid to regulate the expression of lytic proteins [36,37]. This system was then included in C@ECN, denoted as C@ECN-PL. The oxygen-induced disintegration capability of C@ECN-PLs is demonstrated in Figure 1I. Compared to that of C@ECN-PLs under normoxic conditions, the growth of C@ECN under hypoxic conditions decreased, with a decrease of only half at 20 h. However, under hypoxic conditions, the growth of C@ECN-PLs was restricted after 8 h, and lytic proteins were expressed under the Pvhb promoter; thus, there was virtually no increase in cell density. These results indicate that C@ECN-PLs induced the disintegration of ECN specifically under hypoxia, leading to a reduction in cell density within C@ECN-PLs.

### C@ECN-PLs inhibit COX-2 expression in CT26 and MCF-7 tumor cells

**Fig. 2.**
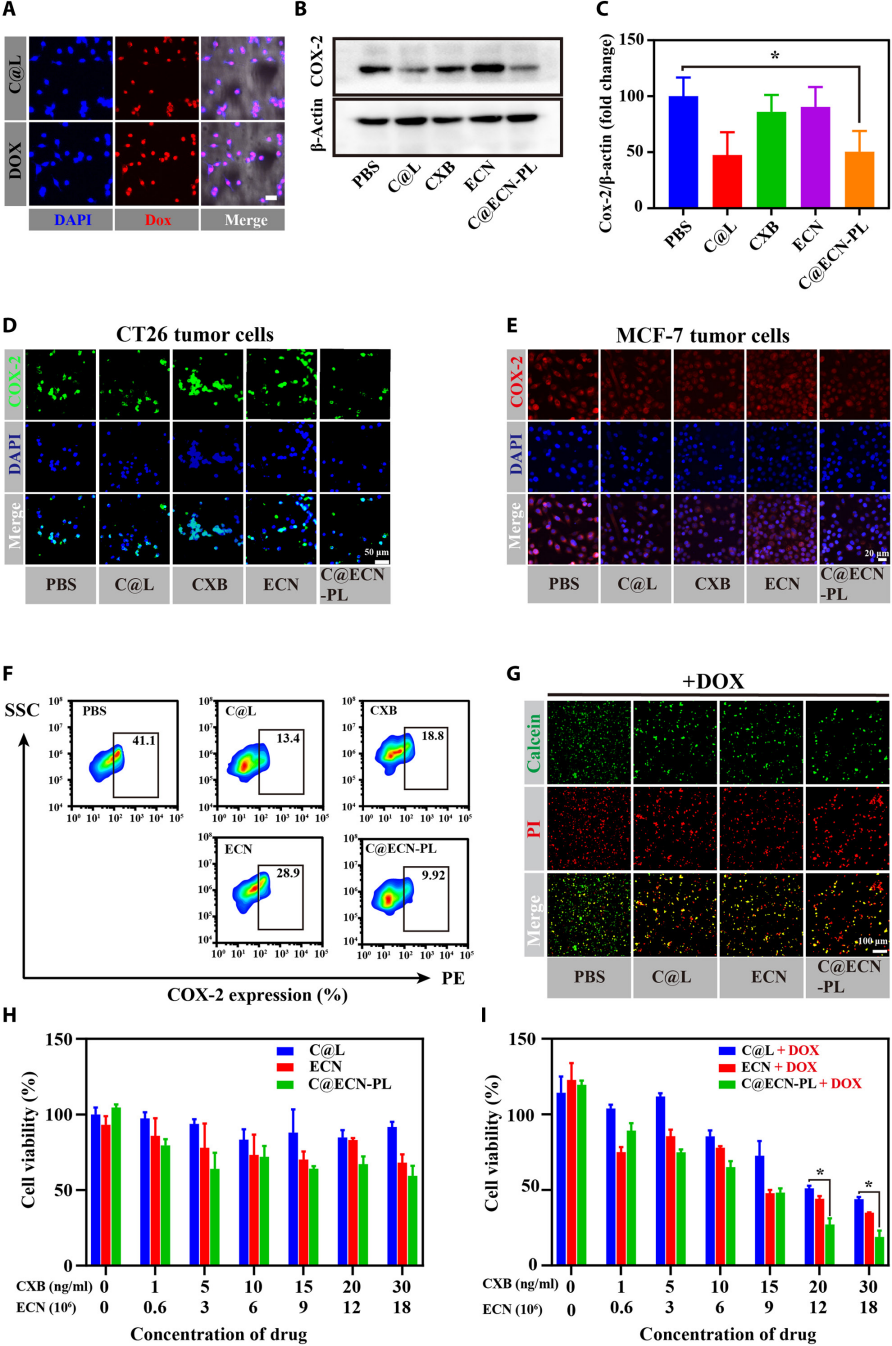
C@ECN-PLs inhibited COX-2 expression and enhance DOX toxicity in vitro. (A) Representative image of cell uptake after incubation with C@L or C@ECN-PL. Scale bars, 50 μm. (B and C) Quantitative analysis of the levels of COX-2 after various treatments with WB analysis. (D and E) Representative image of C@ECN-PLs inhibiting COX-2 expression in CT26 or MCF-7 tumor cells. (F) Quantitative analysis of the level of COX-2 after various treatments by FCM. (G) Representative image of CT26 tumor cells following various treatments without DOX. (H and I) Cell viability of CT26 cells as detected by CCK-8 following various treatments (*n* = 3), at a DOX concentration of 1.5 μM. Statistical analysis was performed using 2-tailed Student’s *t* tests. *P < 0.05.

To evaluate the antitumor capability of C@ECN-PLs, cell uptake experiments using CT26 tumor cells. ICG was used as a fluorescent dye instead of CXB to observe the cellular uptake behavior of CT26 tumor cells. As shown in Fig. 2A, similar fluorescence intensities were observed when CT26 tumor cells were treated with I@L or I@ECN-PLs. These results indicate that C@ECN-PLs can be effectively be taken up by CT26 tumor cells. High COX-2 protein expression always leads to the PGE-2 expression, thereby increasing the drug resistance of tumor cells. Therefore, we chose CXB (a COX-2 inhibitor) to inhibit the expression of COX-2 in tumor cells, thus reducing the drug resistance of tumor cells. The ability of C@ECN-PLs to inhibit COX-2 protein expression was assessed through WB analysis. As shown in Fig. 2B and C, compared with the PBS treatment group, both the C@L and C@ECN-PL treatment groups exhibited 50% inhibition of COX-2 protein expression. In contrast, free CXB showed an inhibitory effect similar to that of the PBS control, possibly due to its poor water solubility, resulting in a decreased cellular uptake capacity [38]. Immunofluorescence (IF) also revealed results similarity to those of WB (Fig. 2D and Fig. S1). To further confirm the inhibitory effect of C@ECN-PL on COX-2, COX-2 expression in MCF-7 cells after different drug treatments was examined. As shown in Figure 2E and S2, the red fluorescence intensity of COX-2 was weaker in the C@L- or C@ECN-PL-treated groups than in the PBS group, indicating the specific inhibition of COX-2 expression in tumor cells by C@ECN-PLs. Quantitative analysis of COX-2 inhibition by C@ECN-PLs was performed using flow cytometry. As shown in Figure 2F, the percentage of COX-2-expressing CT26 cells was 13.4%, 18.8%, and 9.92% after treatment with C@L, CXB, and C@ECN-PLs, respectively, compared to 41.1% in the PBS treatment group. These results further confirmed that C@ECN-PLs effectively inhibited COX-2 expression in tumor cells.

C@ECN-PLs can inhibit COX-2 expression, thereby suppressing the synthesis of PGE-2 and down-regulating the expression of the downstream antiapoptotic gene Bcl-2, reducing tumor cell resistance [39]. Therefore, the antitumor effect of C@ECN-PLs in combination with DOX (1.5 μM) was evaluated using iodine pyridine reagent a CCK-8 assay. As shown in Fig. 2G and Fig. S3, compared to the other treatment groups (without DOX), the combination of C@ECN-PLs and DOX inhibited tumor cell growth by 70%. Consistent results between the IF and CCK-8 assays were maintained (Fig. 2H and I). In contrast, treatment with DOX alone or C@ECN-PL alone did not achieve a therapeutic effect aimilar to that of C@ECN-PL + DOX, indicating that C@ECN-PLs can enhance the sensitivity of CT26 cells to DOX by inhibiting COX-2 expression and improving the therapeutic efficacy of DOX.

### C@ECN-PL enhanced CXB accumulation at the tumor site in vivo

**Fig. 3.**
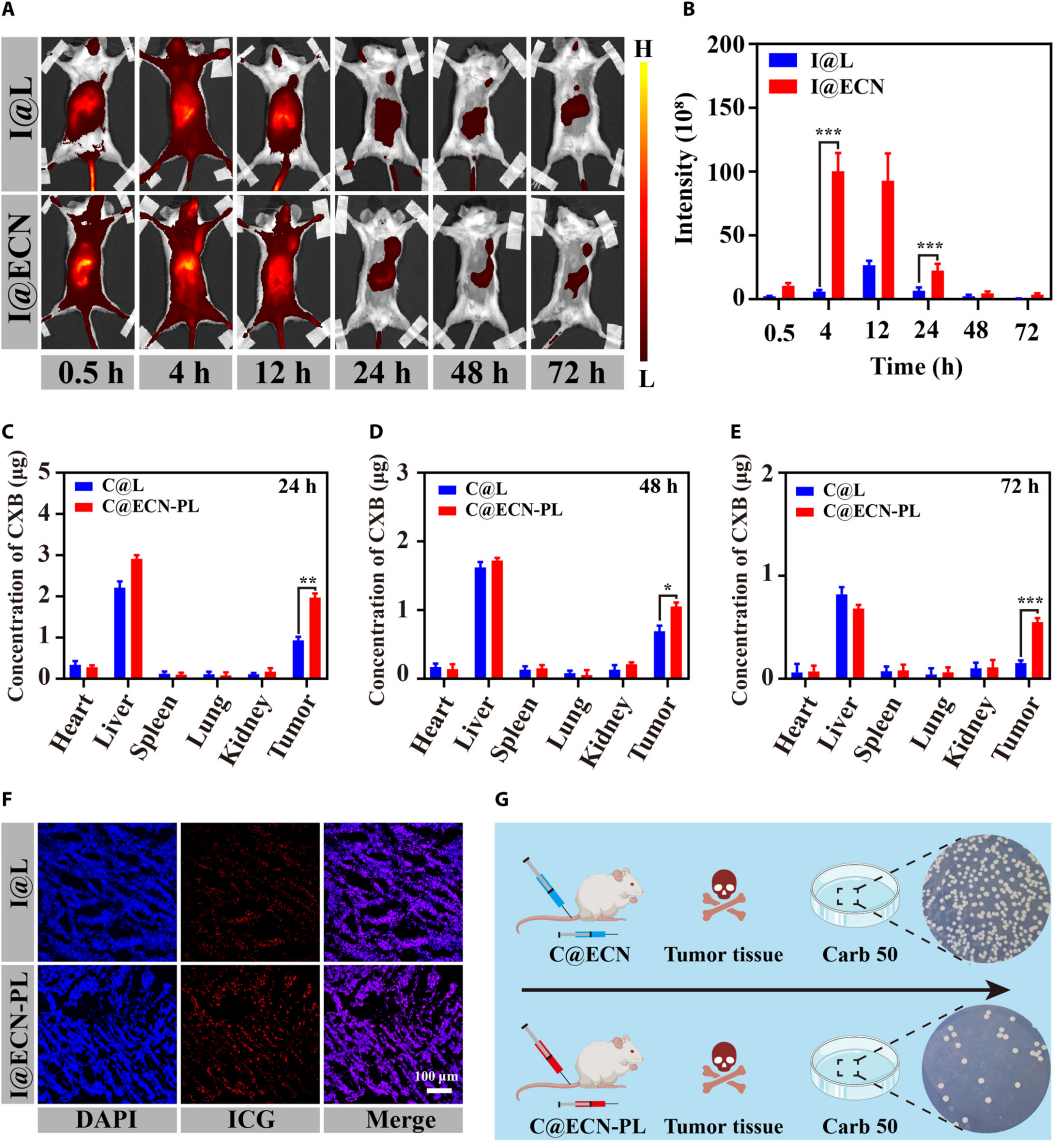
Pharmacokinetics and biodistribution of C@ECN-PLs in vivo. (A) Real-time fluorescence images of CT26-tumor-bearing mice following an intravenous injection of I@L or I@ECN-PLs at different time points. (B) Quantification of the ICG fluorescence signal at the tumor site. (C to E) The concentration of CXB distributed on the main organs and tumor site at different times following various treatments in vivo. (F) Representative images of ICG distribution at the tumor site. Scale bar, 100 μm. (G) Representative images of agar plates containing ECN colonized from the tumor site after treatment with C@ECN or C@ECN-PL. Statistical analysis was performed using 2-tailed Student’s *t* tests. *P < 0.05; **P < 0.01; ***P < 0.001.

To address the potential adverse effects of prolonged usage of CXB on cardiovascular and cerebrovascular health, a tumor-targeting formulation, C@ECN-PL, was developed by loading CXB onto the surface of the ECN. To confirm its active-tumor-targeting ability, an in vivo imaging system was used to examine the biodistribution of C@ECN-PLs in a mouse tumor model. First, we replaced CXB with ICG because ICG emits long-wavelength light (808 nm), making its red fluorescence signal easier to capture. Four hours after intravenous injection, both I@L and I@ECN-PLs exhibited widespread distribution throughout the body (Fig. 3A and B). At 24 h, red fluorescence predominantly accumulated in the tumor and liver regions. By 72 h, the fluorescence intensity at the tumor site following I@ECN-PL treatment was 4-fold greater than that observed with I@L, clearly demonstrating the effective tumor-targeting capability of C@ECN-PL in vivo. In addition, the concentration of CXB in major organs was determined using UV-VIS spectroscopy. As shown in Fig. 3C to E, even after 72 h of treatment, the accumulation of C@ECN-PL at the tumor site was 3 times greater than that achieved with C@L treatment. This finding was further supported by tumor slice analysis, confirming the enhanced drug accumulation by I@ECN-PLs at the tumor site (Fig. 3F and Fig. S4) due to the infiltration and colonization of the ECN. Finally, to ensure the safety of C@ECN-PL in vivo, the oxygen-induced disintegration capability of C@ECN-PL was evaluated. The results shown in Fig. 3G and Fig. S5 demonstrated a significant reduction in the number of ECNs containing the disintegration circuit within C@ECN-PLs compared to the C@ECN treatment group, indicating that C@ECN-PL can selectively induce the disintegration of ECNs at the tumor site, thereby enhancing their safety profile in vivo.

### C@ECN-PLs inhibited the growth of CT26 tumor cells in vivo

**Fig. 4.**
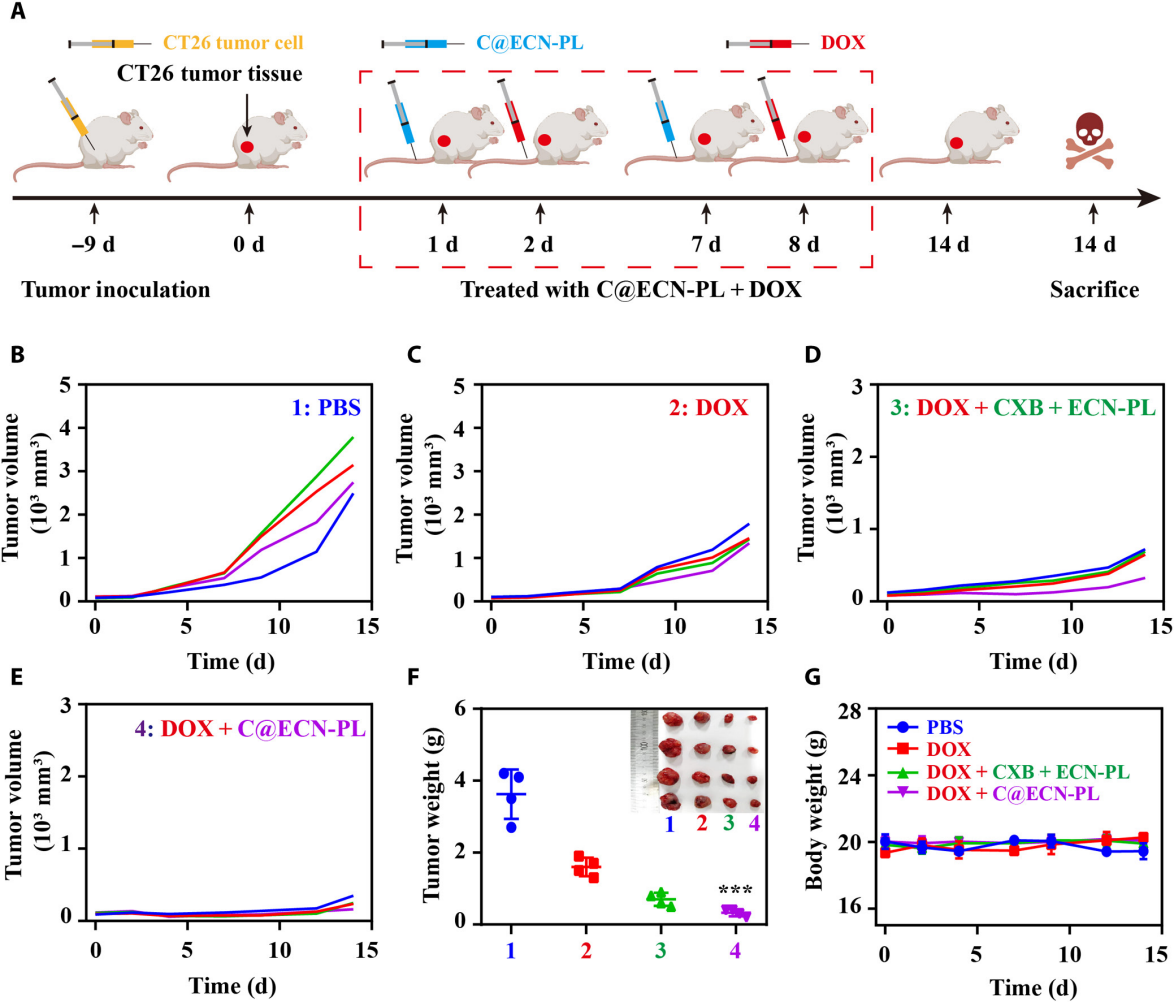
Antitumor efficacy of C@ECN-PLs in CT26 tumor-bearing mice in vivo. (A) Schematic diagram of the anti-CT26tumor experimental design. (B to E) Tumor growth curves of CT26 tumor-bearing mice after treatment (n = 4). (F) Weights and photos of excised CT26 tumors after treatment for 14 d (*n* = 4). (G) Body weight curves of CT26-tumor-bearing mice after being treated with different treatment (*n* = 4). The Data are presented as the means ± SDs.

The in vivo antitumor efficacy of C@ECN-PLs was evaluated using a CT26 tumor-bearing mouse model (Fig. 4A). As depicted in Fig. 4B to E, during the 14-day treatment period, DOX, CXB + ECN-PL + DOX, and C@ECN-PL + DOX showed tumor growth inhibition rates of approximately 50%, 70%, and 84%, respectively, compared to the PBS control group. After the completion of the treatment (Fig. 4F), the C@ECN-PL + DOX group exhibited a significantly reduced tumor mass of 0.3 ± 0.1 g, while the PBS group had a tumor mass of 3.7 ± 0.3 g. No significant changes in body weight were observed during the treatment (Fig. 4G), suggesting that C@ENC-PLs inhibited tumor growth.

### C@ECN-PLs inhibited COX-2 expression and enhanced T lymphocyte infiltration in CT26 tumor model

**Fig. 5.**
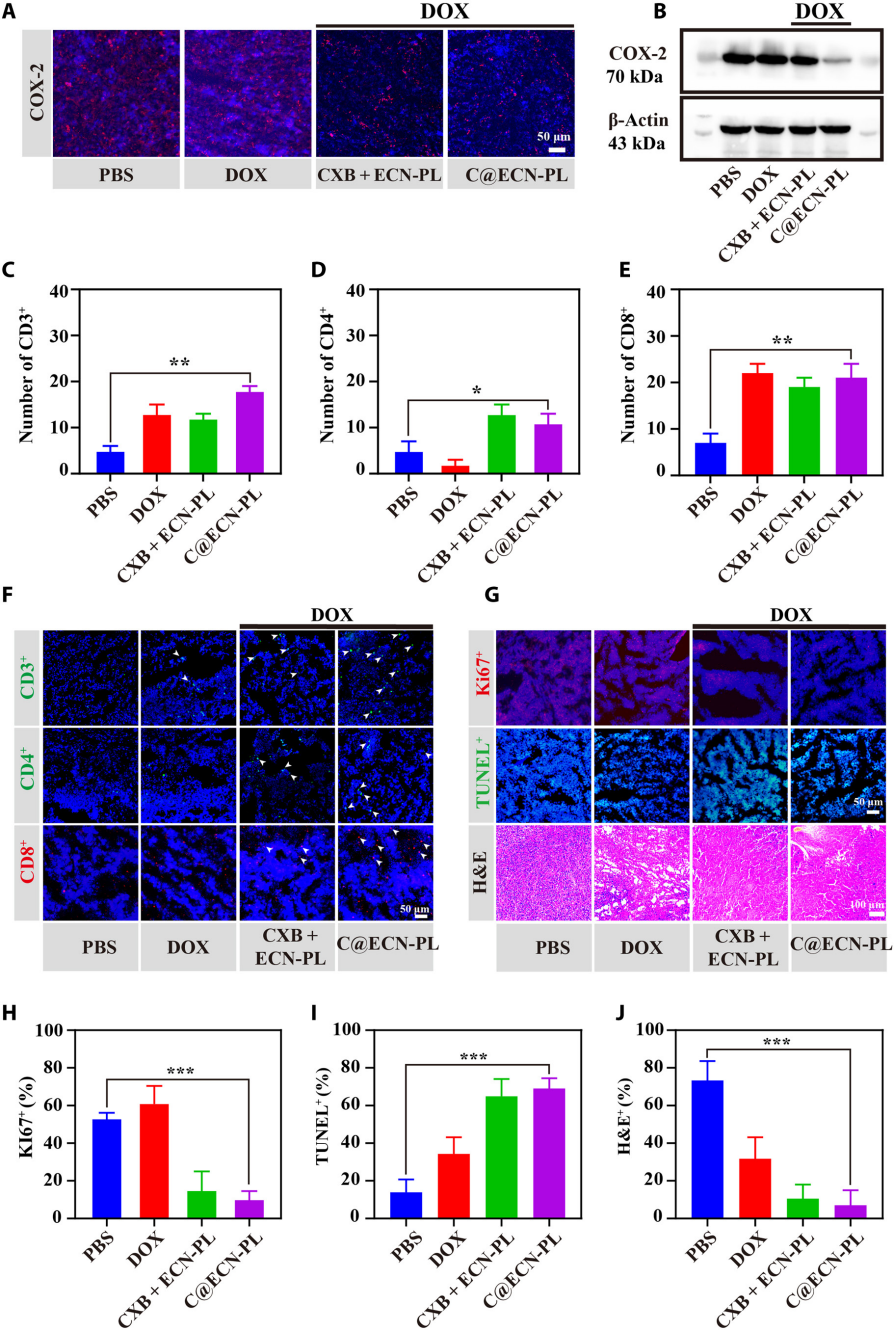
Effects of C@ECN-PLs on T-cell infiltration and the induction of CT26 cells apoptosis in vivo. (A and B) The expression level of COX-2 in CT26-tumor-bearing mice after treatment in various groups. (C to E) Quantification of CD3^+^, CD4^+^, and CD8^+^ T cells in CT26 tumor slices. (F) Representative fluorescence images of CD3^+^, CD4^+^, and CD8^+^ T cells in CT26 tumor slices. Scale bar, 50 μm. (G) Representative images of Ki67 staining, TUNEL staining, and H&E staining. Scale bar, 100 μm. (H to J) Quantification of Ki67^+^, TUNEL^+^, and H&E^+^ staining in CT26 tumor slices. The data are presented as the means ± SDs. Statistical analysis was performed using 2-tailed Student’s *t* tests. *P < 0.05; **P < 0.01; ***P < 0.001.

To determine the antitumor mechanism of C@ECN-PLs, we confirmed the expression of COX-2 in mouse tumor tissues after C@ECN-PL treatment using IF and WB. The results shown in Fig. 5A and Fig. S6 indicate that the red fluorescence after C@ECN-PLs treatment was significantly weaker than that in the other treatment groups. This is because C@ECN-PLs can specifically deliver CXB to the tumor site and inhibit the expression of COX-2. Western blotting was used for further validation (Figure 5B and S7), and the results were consistent with the immunofluorescence results. After C@ECN-PL treatment, the expression of COX-2 in tumor tissues was significantly inhibited. Previous studies have suggested that ECN can serve as an immune adjuvant and interact with the host immune system to activate the cytotoxic T lymphocyte activity and enhance the immune antitumor effects [1,2]. Tumor tissue sections were collected and used to detect CD3^+^, CD4^+^, and CD8^+^ positive cytotoxic T lymphocytes. As shown in Fig. 5C to F, compared to the PBS treatment group, the DOX, CXB + ECN-PL + DOX, and C@ECN-PL + DOX treatment groups exhibited strong immune cell infiltration, with greater numbers of CD3^+^, CD4^+^, and CD8^+^ T lymphocytes in the DOX treatment group than the PBS treatment group. However, the DOX treatment group had a lower number than the C@ECN-PL + DOX treatment group. This may be because DOX only induces immunogenic cell death in tumor cells, leading to T lymphocyte infiltration. C@ECN-PL + DOX inhibited the expression of COX-2, thereby reversing the suppressive effect on the tumor immune microenvironment and enhancing T-cell infiltration. Finally, tumor sections were detected using TUNEL or KI67 detection tools. KI67 is an indicator of cell proliferation capacity. As shown in Fig. 5G to J, the red fluorescence in the PBS treatment group accounted for approximately 65 ± 3%, while the percentage of Ki67-positive cells after C@ECN-PL+DOX treatment was less than 10%. TUNEL staining, which detects tumor cell apoptosis, yielded similar results. The percentage of TUNEL-positive tumor cells after C@ECN-PL+DOX treatment was approximately 70%, which is approximately twice as high as that in the PBS or DOX treatment groups. Finally, H&E staining revealed extensive lysis of tumor cells after C@ECN-PL+DOX treatment. These results indicate that C@ECN-PL+DOX can achieve its antitumor effect by inhibiting tumor cell proliferation and inducing tumor cell apoptosis.

### Biosafety of C@ECN-PLs in vivo

**Fig. 6.**
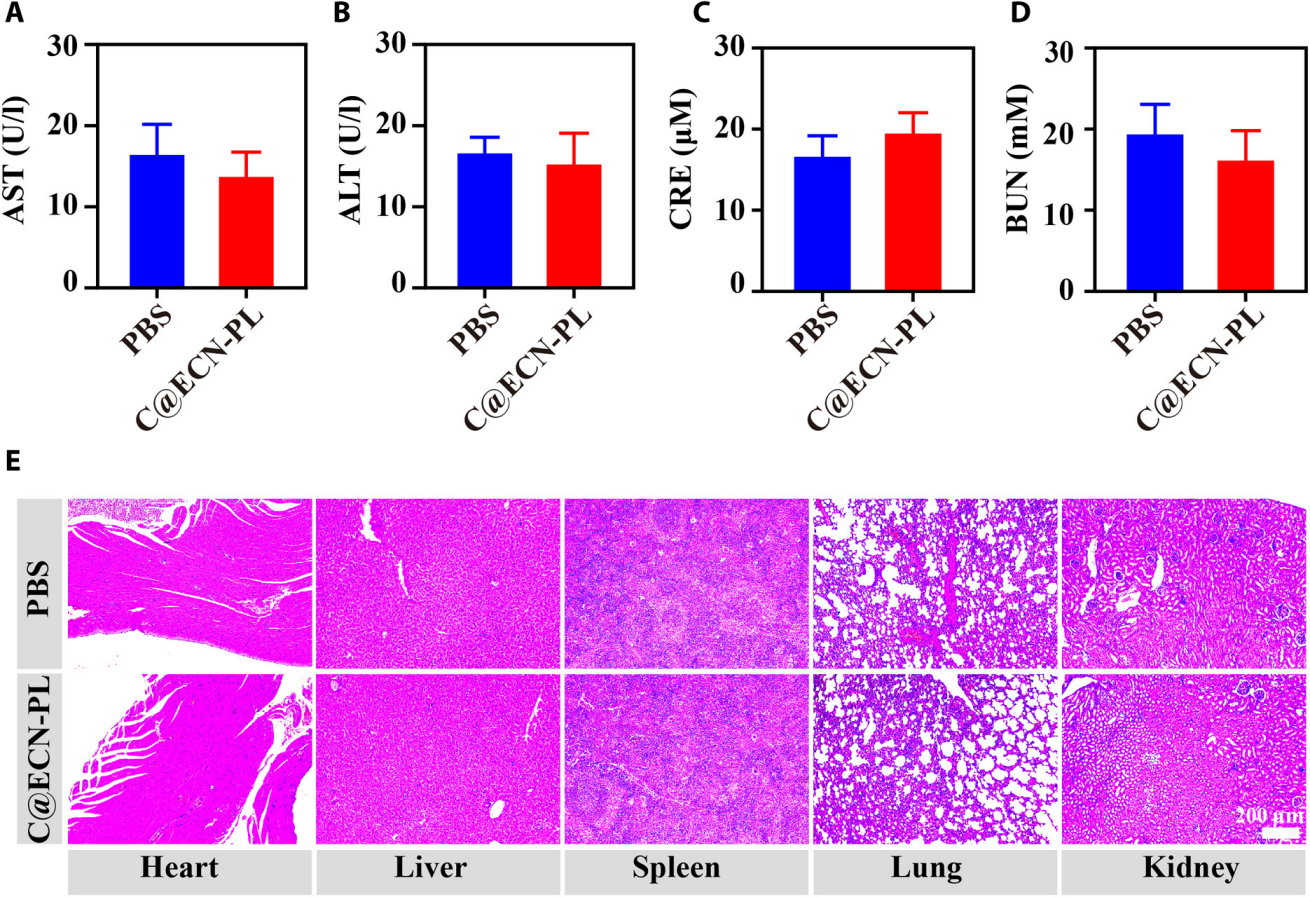
Biosafety tests of C@ECN-PLs in BALB/c mice. (A and B) Serum biochemistry data of AST and ALT represent the liver function (*n* = 3). (C and D) Serum biochemical data for BUN and CRE indicate kidney function (*n* = 3). (E) H&E staining images of the major organs on day 14. Scale bar, 100 μm. The data are presented as the means ± SDs.

The biosafety of C@ECN-PLs was evaluated in mice by monitoring their renal and liver functions and conducting H&E staining of their major organs. Liver function biomarkers, such as aspartate aminotransferase (AST) and alanine aminotransferase (ALT), were assessed to determine potential liver toxicity. Fig. 6A and B, shows that the levels of AST and ALT remained unaffected after 14 d of treatment with C@ECN-PL + DOX. The same results were also obtained when evaluating kidney function markers, including blood urea nitrogen (BUN) and creatinine (CRE). The data presented in Fig. 6C and D, confirmed that the administration of C@ECN-PL + DOX did not cause any toxicity or impairment of renal function. Moreover, histological examination using H&E staining compared the normal tissues of mice treated with PBS to those of mice treated with C@ECN-PL + DOX (Fig. 6E). These findings indicate that C@ECN-PLs do not exhibit significant long-term toxicity in normal tissues and supports their potential as an ideal candidate for tumor treatment due to their favorable biosafety profile.

## Conclusion

**Fig. 7.**
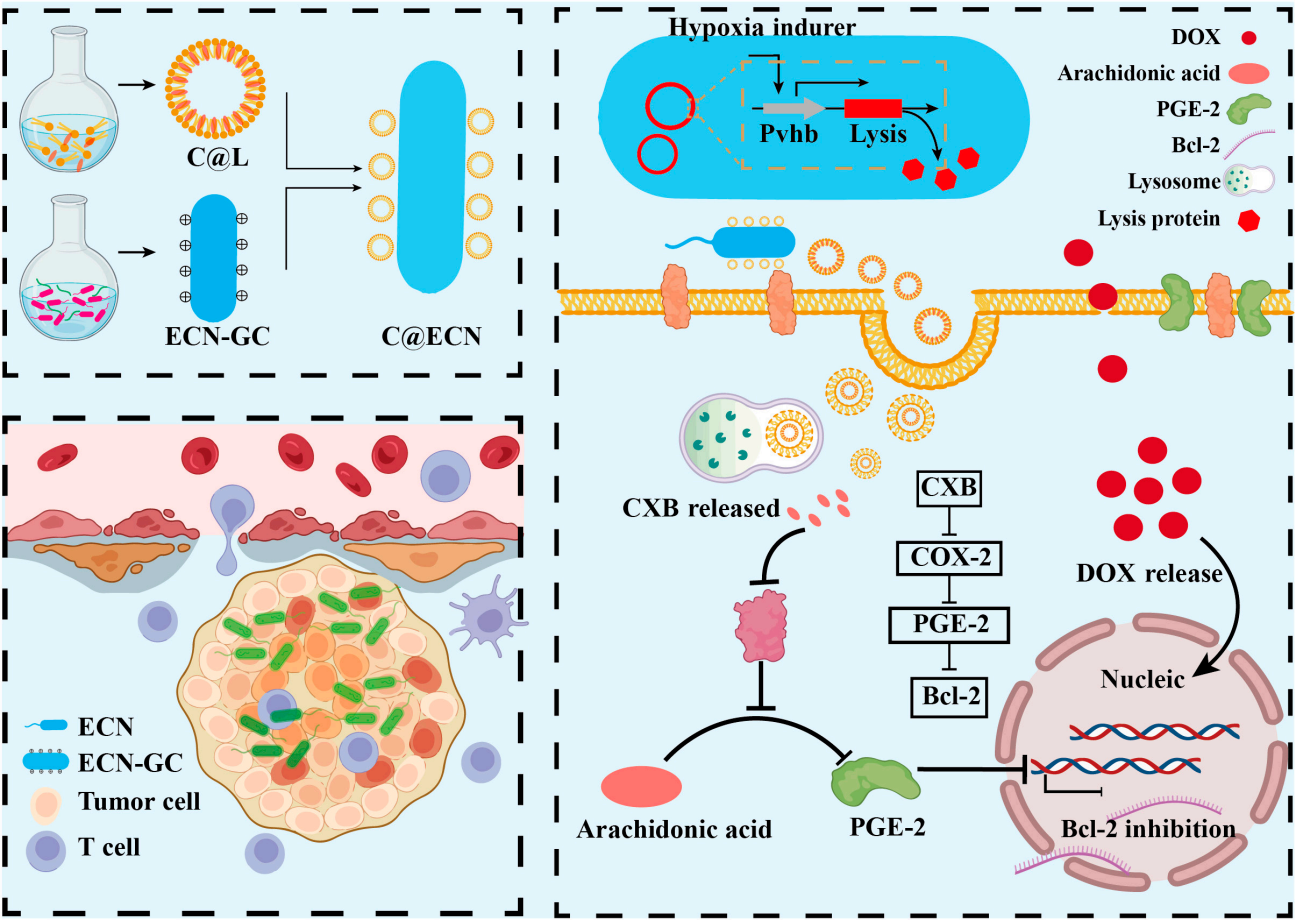
Schematic illustration of the synthesis route of C@ECN-PL and the mechanism of enhancing the antitumor effect by inhibiting COX-2 and downstream prostaglandin E2 (PGE-2) expression and facilitating T cell infiltration in the tumor microenvironment.

Tumor cells displaying an up-regulated COX-2/Bcl-2 axis demonstrate heightened resistance to apoptosis and diminished effectiveness of chemotherapy medications. CXB has been identified as a specific inhibitor of COX-2 and can selectively suppress the inflammatory process at tumor sites. However, the potential vascular toxicity of CXB limits its prolonged use in vivo [40]. To mitigate the toxic effects of CXB on the body, a tumor-targeted drug delivery strategy called C@ECN-PLs was proposed (Fig. 7). C@ECN-PLs actively deliver CXB to tumor sites based on the inherent tumor-targeting ability of the ECN. C@ECN-PLs not only reduced the toxicity and adverse effects of CXB but also enhanced the sensitivity of tumor cells to DOX by inhibiting the expression of COX-2, thereby limiting the COX-2/PGE-2 pathway. C@ECN-PLs selectively colonized tumor sites and effectively suppressed COX-2 expression in tumor cells (Figs. 2, 3, and 5). Considering the safety of C@ECN-PLs, the hypoxia-induced circuit, pGEX-Pvhb-Lysis, was introduced into the ECN to facilitate the elimination of C@ECN-PL from the body (Figs. 1 and 3). Furthermore, considering that ECN can act as an immune adjuvant to recruit immune cells to infiltrate tumor sites, CD3^+^, CD4^+^, and CD8^+^ immune cells accumulate in the tumor microenvironment, reversing tumor immune suppression (Fig. 5) [41]. C@ECN-PLs demonstrates excellent biocompatibility in vivo (Fig. 6).

Currently, ECN-based tumor treatment strategies have attracted increasing attention. Although engineered bacteria expressing therapeutic factors have shown promising tumor treatment effects, the inflammatory response and in vivo safety associated with ECNs remain crucial issues to be addressed. Therefore, a tumor-targeted drug delivery strategy in which liposomes are electrostatically adsorbed onto the surface of ECN was proposed for the delivery of various drugs, including hydrophilic and hydrophobic drugs. The introduction of the pGEX-Pvhb-Lysis circuit ensures its safety in vivo. However, surface-adsorbed liposomes may limit the activity of ECNs due to their size (90 to 100 nm), and the pGEX-Pvhb-Lysis circuit requires the maintenance of antibiotics, which is not conducive to long-term storage. In summary, C@ECN-PLs serve as an excellent drug delivery platform for a variety of drugs, including small molecules, proteins, and RNA, among others.

## Acknowledgments

We thank C. Liu (Xi’an Jiao Tong University) for the gift of ECN strain. We also thank the Research Center of Analysis and Test (ECUST) for help with data collection and characterization. We thank F. Chen (JXNU), X. Chen, R. Wang, and F. Gao (ECUST) for help and comments.

**Funding:** This work was supported by the National Key Research and Development Program of China (grant no. 2020YFA0908900), the Natural Science Foundation of Shanghai (grant no. 22ZR1416000), the Fundamental Research Funds for Central Universities, and the Open Funding Project of State Key Laboratory of Microbial Metabolism (grant no. MMLKF24-01).

**Author contributions:** N.J.: Investigation and writing—original draft. W.D.: Data analysis. X.Z., J.C., and L.Y.: Experimental operation. X.Y. and Y.Z.: Platform support. J.Q.: Platform support and writing—review. J.H.: Supervision, funding acquisition, and writing—review and editing.

**Competing interests:** The authors declare that they have no competing interest. The authors have filed a patent based on this work (CN/202410409102.2).

## Data Availability

The data are available from the authors upon reasonable request.

## Supplementary Materials

Supplementary 1 Figs. S1 to S7
