# Supplementary material for: Lipid-Encapsulated Engineered Bacterial Living Materials Inhibit Cyclooxygenase II to Enhance Doxorubicin Toxicity
Source: Biodes Res. 2024 Jun 25;6:0038. doi: 10.34133/bdr.0038 (PMC11197476; doi:10.34133/bdr.0038)
Supplement: Supplementary 1 — Figs. S1 to S7 [file bdr.0038.f1.docx]

**Lipid-encapsulated Engineered Bacteria Living Materials Inhibit COX-2 to enhance DOX Toxicity**

*Ning Jiang ^a, b^,Wanqing Ding ^a, b^, Xiaojuan Zhu ^a, b^**, Jianshu Chen ^a, b^, Lin Yang ^c^, Xiaoping Yi ^a^, Yingping Zhuang ^a, b^, Jiangchao Qian ^a^, Jiaofang Huang ^a, c^ **

a State Key Laboratory of Bioreactor Engineering, East China University of Science and Technology, Shanghai 200237, China

b Shanghai Collaborative Innovation Center for Biomanufacturing (SCICB), East China University of Science and Technology, Shanghai 200237, China

c College of Life Science, Jiangxi Normal University, Nanchang 330022, China


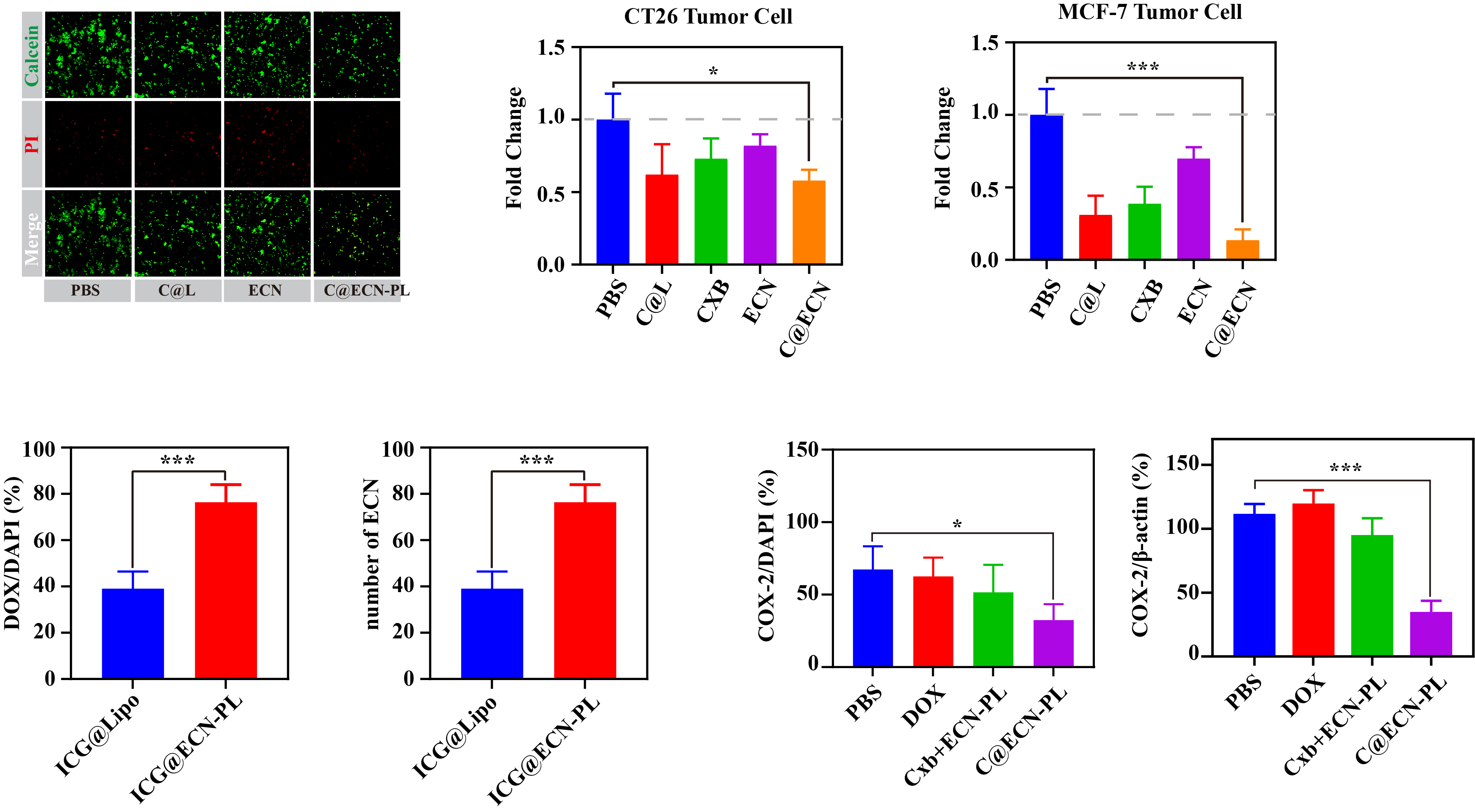


**Figure S1:** Quantitative analysis the level of COX-2 after various treated by immune flourescence in CT26.


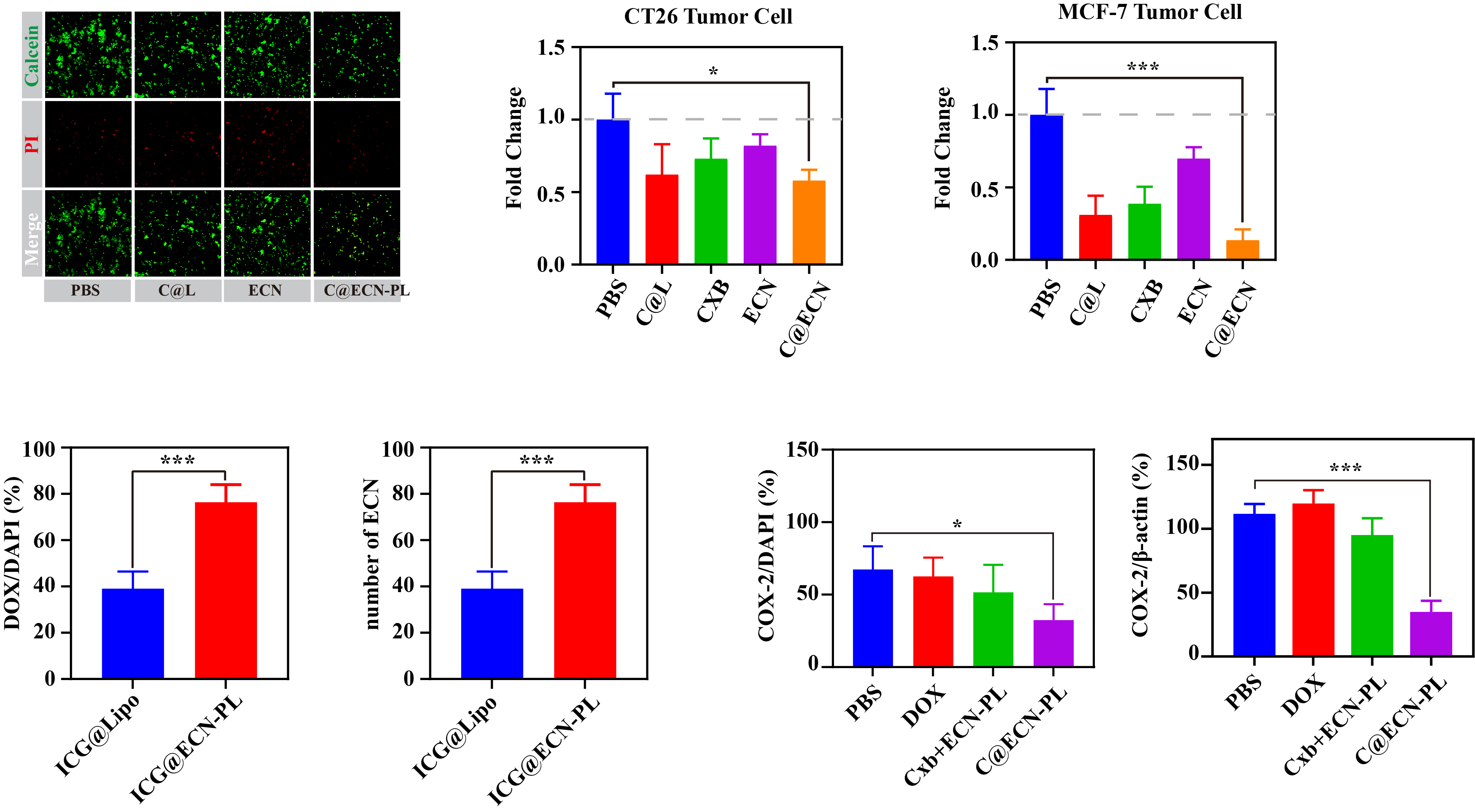


**Figure S2:** Quantitative analysis the level of COX-2 after various treated by immune flourescence in MCF-7.


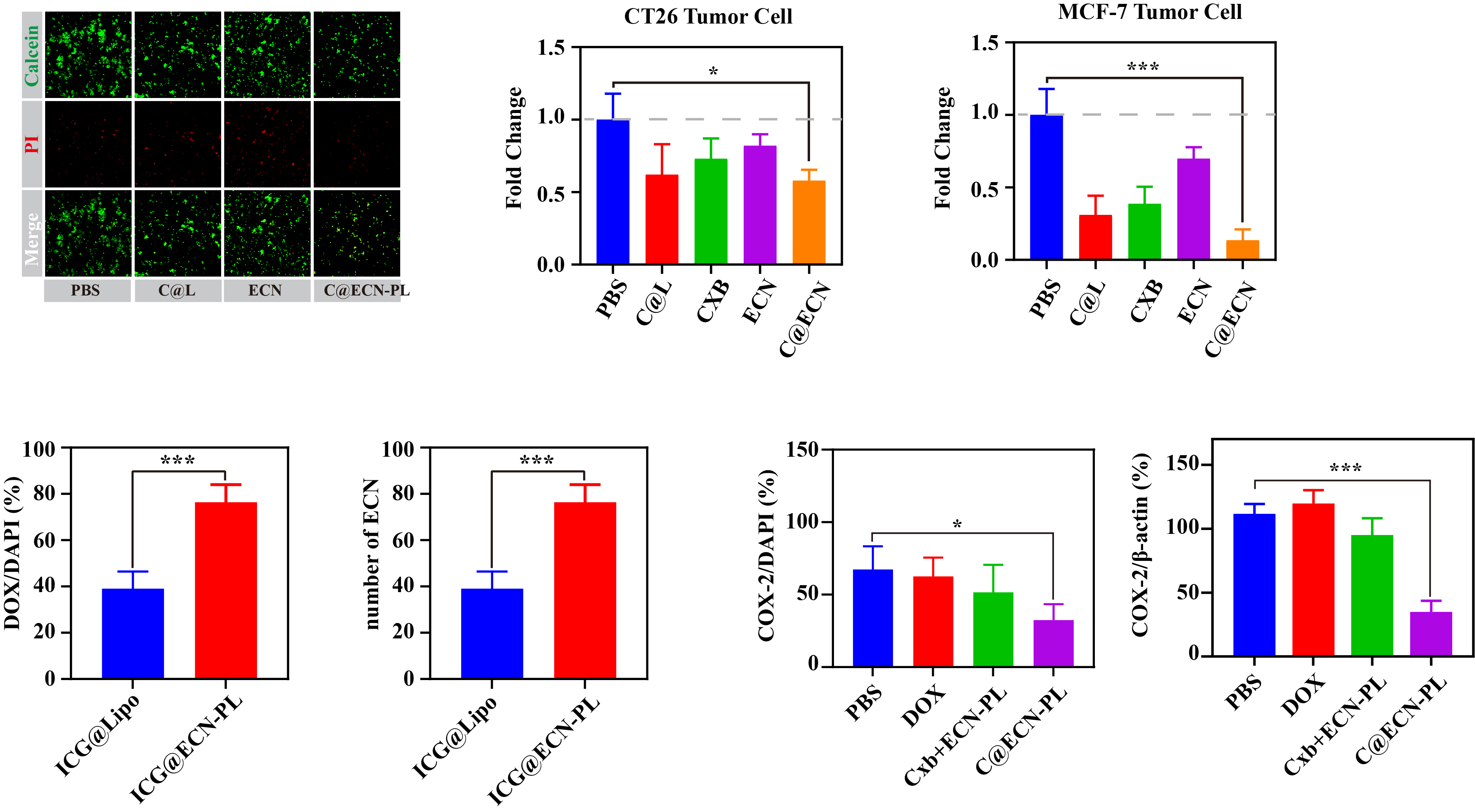


**Figure S3:** Representative image of CT26 tumor cells following various treatments without DOX.


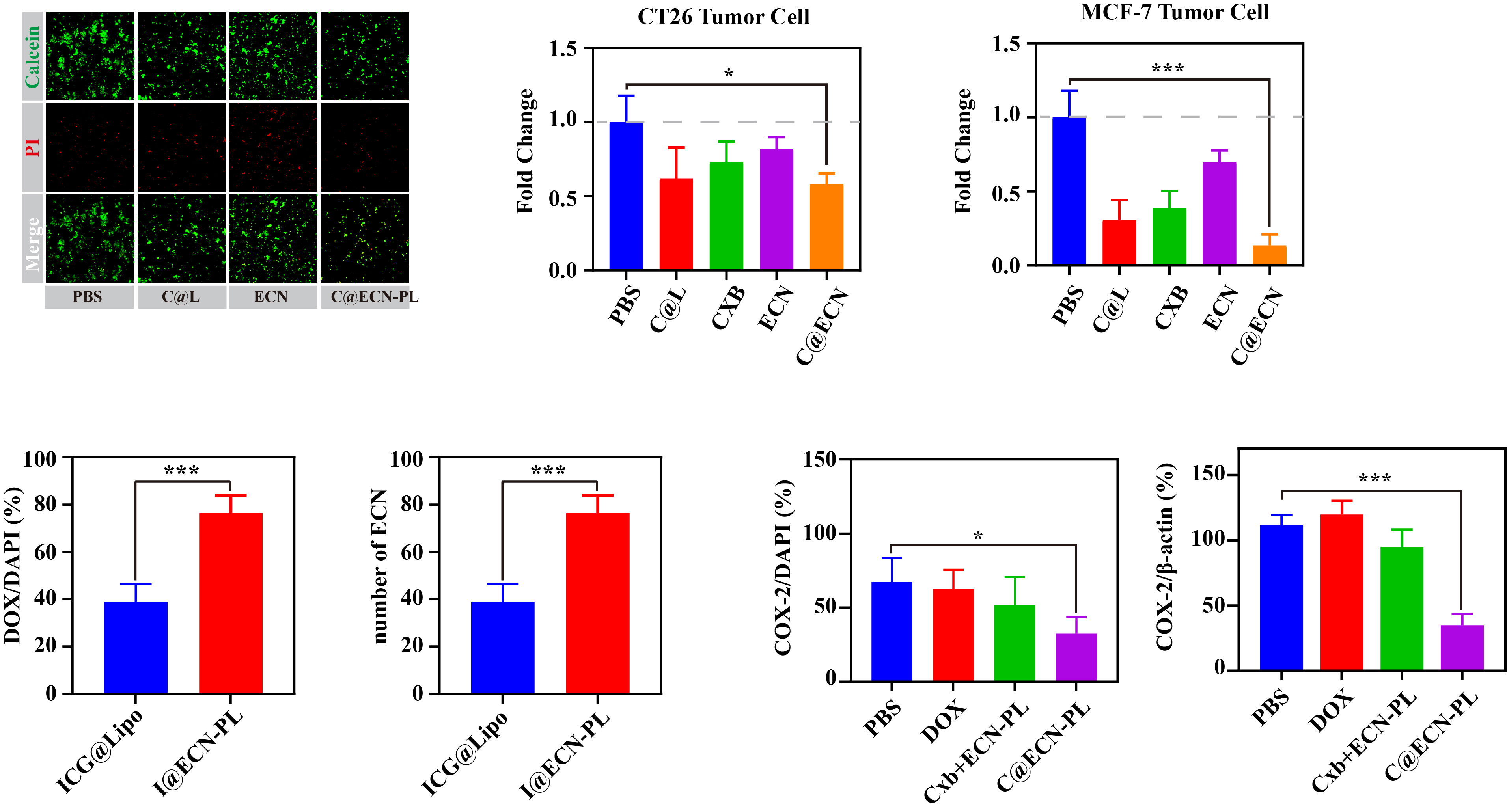


**Figure S4:** Quantitative analysis the level of ICG after various treated by immune flourescence.


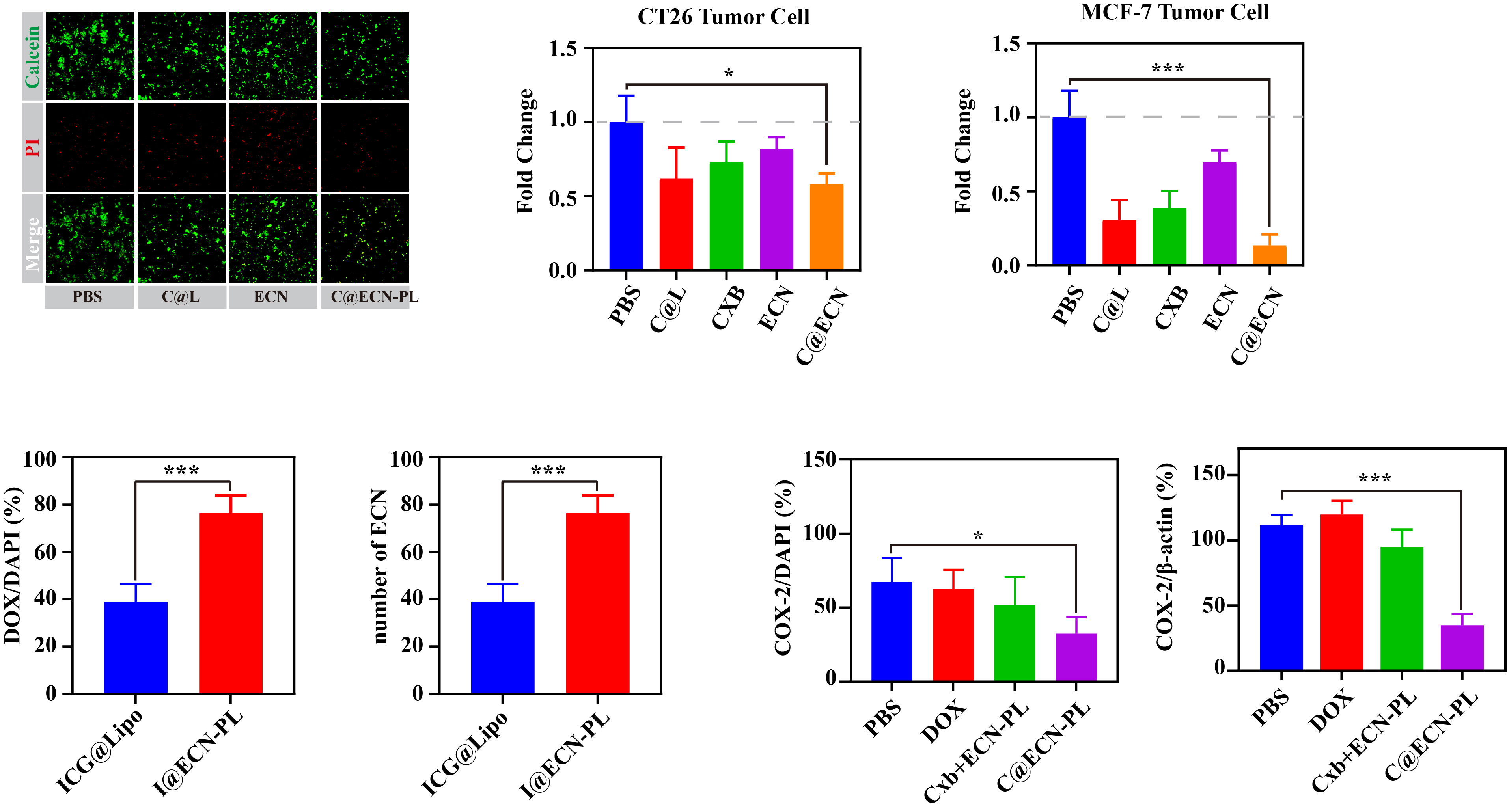


**Figure S5:** Quantitative analysis the level of ECN after treated with C@ECN or C@ECN-PL, respectively.


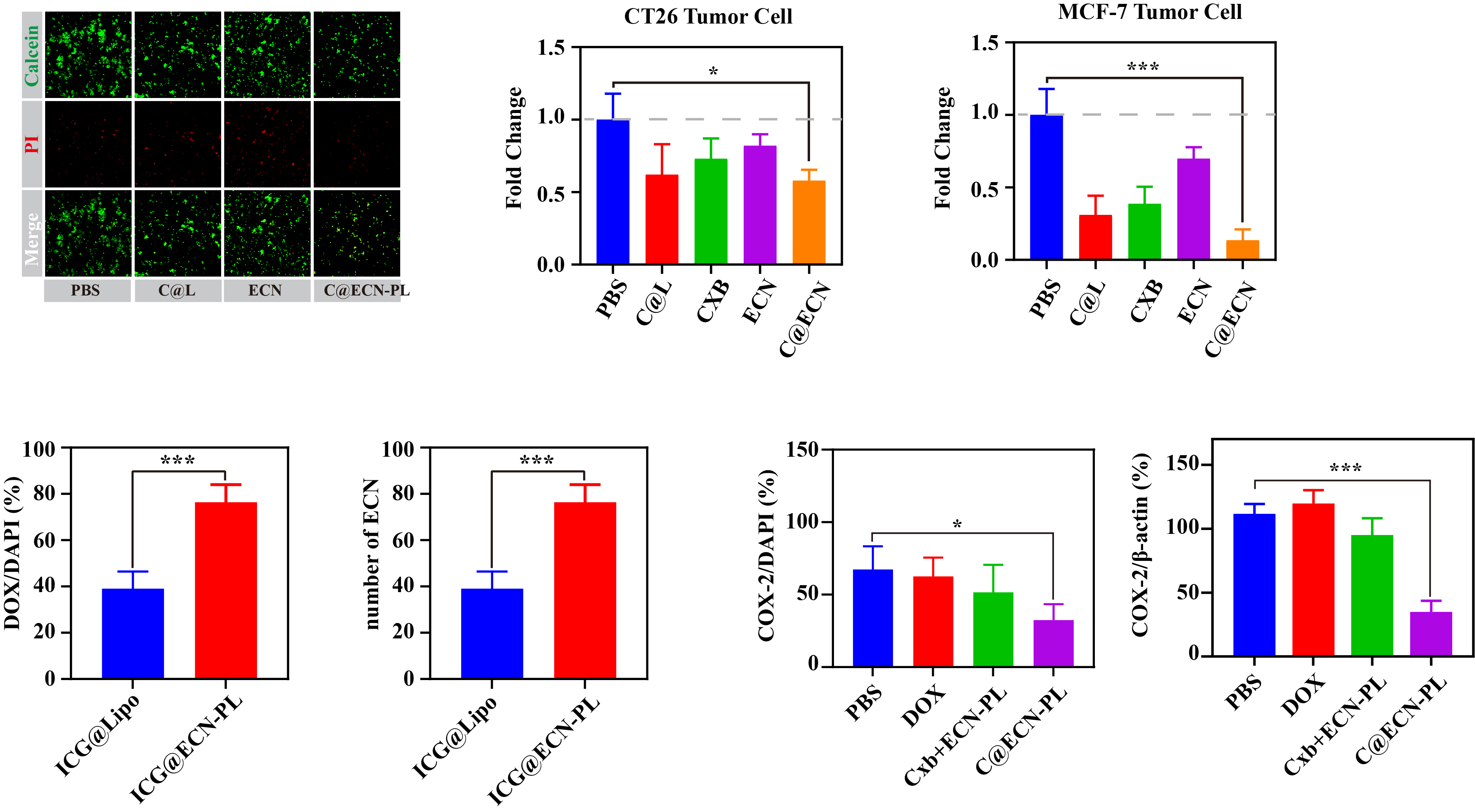


**Figure S6:** Quantitative analysis of COX-2 levels following various *in vivo* treatments using immune flourescence.


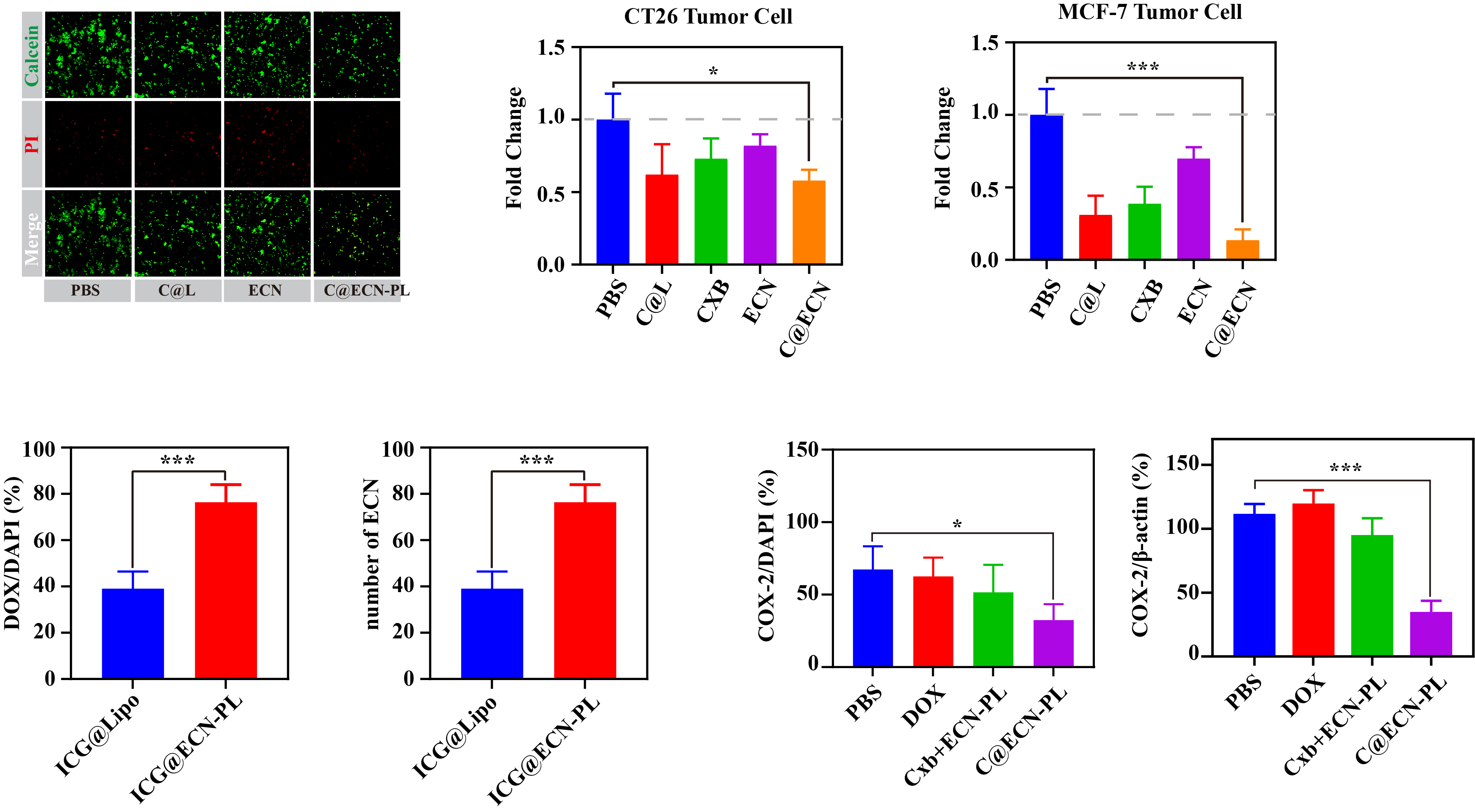


**Figure S7:** Quantitative analysis of COX-2 levels after various treatments by western blot *in vivo*.
